# Ameliorative Effect of Ginsenoside Rc on 5-Fluorouracil-Induced Chemotherapeutic Intestinal Mucositis via the PI3K-AKT/NF-κB Signaling Pathway: In Vivo and In Vitro Evaluations

**DOI:** 10.3390/ijms252313085

**Published:** 2024-12-05

**Authors:** Liyue Xu, Xiaolan Zhao, Fei Tang, Jingnan Zhang, Cheng Peng, Hui Ao

**Affiliations:** 1 State Key Laboratory of Southwestern Chinese Medicine Resources, Chengdu University of Traditional Chinese Medicine, Chengdu 611137, China; 2 Innovative Institute of Chinese Medicine and Pharmacy, Chengdu, University of Traditional Chinese Medicine, Chengdu 611137, China

**Keywords:** ginsenoside Rc, 5-Fu, chemotherapeutic intestinal mucositis, PI3K-AKT, NF-κB, anti-inflammatory, anti-apoptotic

## Abstract

5-Fluorouracil (5-Fu) is a chemotherapeutic agent widely used to treat various cancers, which causes intestinal mucositis as a common side effect. Ginsenoside Rc, an active compound with anti-inflammatory, antioxidant, immunomodulatory, and antitumor properties, has protective effects against chemotherapy-induced mucositis caused by 5-Fu. This study aims to evaluate the protective effects of Rc on 5-Fu-induced chemotherapy-related mucositis and to elucidate its underlying mechanisms. In vivo experiments were conducted to measure intestinal permeability and assess the effects of Rc on body weight loss, diarrhea, and intestinal pathology induced by 5-Fu. Network pharmacology was also employed to explore potential mechanisms. In vitro, IEC-6 cell models were used to validate the cytoprotective effects of Rc, including assessments of cell viability, apoptosis, lactate dehydrogenase (LDH) release, and changes in inflammatory cytokine levels. The results indicate that Rc significantly ameliorated body weight reduction, diarrhea, and intestinal damage in mice treated by 5-Fu. Rc significantly mitigated 5-Fu-induced cellular damage by reducing levels of inflammatory cytokines such as IL-1β, IL-6, and TNF-α and decreasing apoptosis and cell permeability. Western blot analysis revealed that Rc upregulated the expression of Bcl-2 and tight junction proteins and downregulated the expression of Bax. Furthermore, Rc exerts anti-inflammatory and anti-apoptotic effects through PI3K-AKT and NF-κB signaling pathways. In conclusion, ginsenoside Rc demonstrated significant protective effects against 5-Fu-induced intestinal mucositis via the PI3K-AKT/NF-κB signaling pathway, suggesting its potential as a therapeutic agent for chemotherapy-related mucositis.

## 1. Introduction

Chemotherapy is a major treatment for cancer that can be used as an adjuvant therapy in combination with other therapies, such as surgery and radiation therapy, to improve the efficacy of treatment [1,2,3]. However, chemotherapy drugs have drawbacks, as many cannot accurately differentiate between tumor cells and normal cells, potentially causing damage to healthy tissues [4]. 5-Fluorouracil (5-Fu) is an anticancer chemotherapeutic drug that can cause damage to the gastrointestinal tract. Intestinal epithelial cells (IECs), which proliferate rapidly and interact with intestinal microorganisms, are particularly susceptible to the effects of antitumor drugs. The intestinal disorders induced by chemotherapeutic agents are referred to as chemotherapeutic intestinal mucositis (CIM) [5]. In clinical practice, the incidence of CIM is as high as 40%–76% [6]. Symptoms of CIM typically include diarrhea, abdominal pain, and disturbances in the intestinal microbiota. There is currently no specific treatment for CIM. Clinically, ranitidine, octreotide, and omeprazole are commonly used, but they can only alleviate symptoms and have poor efficacy [7]. Therefore, there is an urgent need to develop drugs that can manage CIM.

Our previous studies have shown that a combination of ginsenosides and Atractylodes macrocephala volatile oil could alleviate 5-Fu-induced diarrhea [8]. HPLC has demonstrated that ginsenoside Rc (Rc) is the main ingredient of ginsenosides, which indicates that Rc might alleviate CIM. Notably, Rc is a major active component of ginseng, which is known for its significant pharmacological effects, especially its antioxidant and anti-inflammatory activities [9]. Previous studies have shown that Rc can reduce inflammation levels in cardiomyocytes and repair cellular damage, and it may also be useful in the treatment of metabolic syndrome [10,11]. Moreover, Rc alleviates dextran sulfate sodium (DSS)-induced ulcerative colitis, intestinal inflammation, and barrier dysfunction [12]. However, it remains unknown how or if RC alleviates CIM, which hinders the development and application of anti-CIM drugs.

In this study, in vivo experiments were performed to investigate whether Rc has a therapeutic effect on 5-Fu-induced intestinal injury in mice. Network pharmacology was employed for pathway prediction, followed by in vitro cell experiments and molecular biology tests for validation. This research provides a new potential strategy and theoretical basis for the treatment of CIM.

## 2. Results

### 2.1. Therapeutic Effect of Ginsenoside Rc on 5-Fu-Induced Chemotherapeutic Intestinal Mucositis in Mice

**Figure 1:**
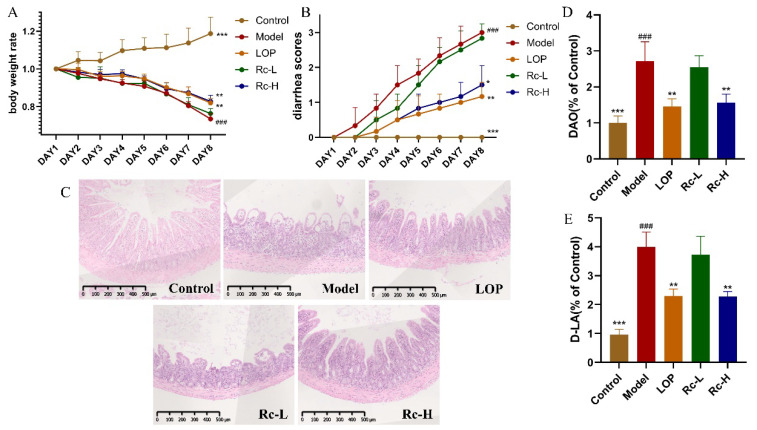
Therapeutic effect of ginsenoside Rc on 5-Fu-induced CIM mice. (**A**) Daily weight changes of mice during the experiment. (**B**) Daily diarrhea of mice during the experiment. (**C**) Representative pictures of HE-stained sections (5×) of mouse ileum. (**D**) DAO content of mice in each group. (**E**) D-LA content of mice in each group. * *p* < 0.05, ** *p* < 0.01, *** *p* < 0.001 vs. model, ### *p* < 0.001 vs. control.

As shown in Figure 1A, the mice began to lose weight after the 5-Fu injection. Until the end of the experiment, the body weight of the model group decreased significantly (*p* < 0.001, Figure 1A) compared with that of the control group, although LOP and 20 mg/kg of Rc significantly reversed this decreasing trend (*p* < 0.01, Figure 1A). There was no difference between the Rc-L group and the model group. The severity of diarrhea in the model group increased with the duration of 5-Fu administration (*p* < 0.01, Figure 1B). The diarrhea was significantly alleviated in the LOP group (*p* < 0.01, Figure 1B) and the Rc-H group (*p* < 0.05, Figure 1B) compared to the model group, while there was no such trend in the Rc-L group.

As shown in Figure 1C, the results of HE sections indicated that 5-Fu induced significant pathological changes in the mouse ileum. Compared with the control group mice, the model group showed epithelial vacuolization, inflammatory cell infiltration, mucosal defects, and crypt damage. The LOP and Rc-H groups effectively suppressed these pathological changes in the model group, although they still had localized damage to the intestinal epithelium. There were no significant changes in the Rc-L group compared with the model group.

We evaluated the intestinal permeability of mice using DAO and D-LA kits. The results are shown in Figure 1D,E. The release of DAO and D-LA significantly increased in the model group compared with the control group, which indicates a significant increase in intestinal permeability in the mice in the model group (*p* < 0.001, Figure 1D,E). This trend was significantly reversed in the LOP and Rc-H groups (*p* < 0.01, Figure 1D,E), while it did not change significantly in the Rc-L group. These results showed that the 5-Fu-induced increase in intestinal permeability in mice was significantly ameliorated in the LOP and Rc-H groups.

### 2.2. Potential Targets of Ginsenoside Rc and Chemotherapeutic Intestinal Mucositis

**Figure 2:**
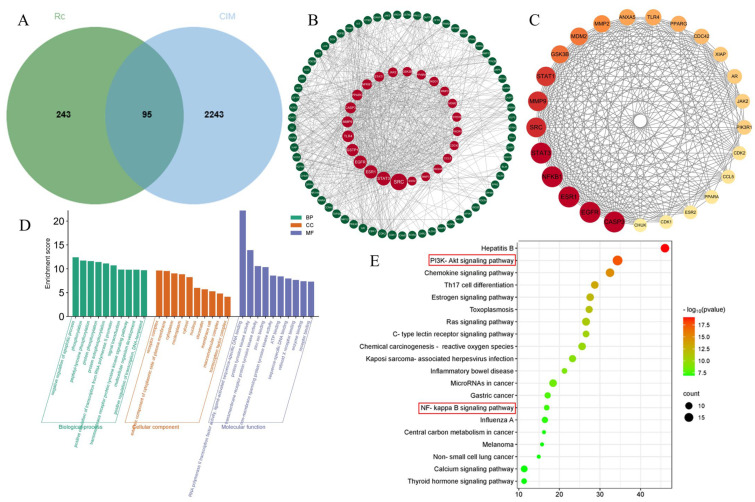
Network pharmacology results. (**A**) VENN plot of Rc-treated CIM. (**B**) PPI plot of Rc interactions with CIM proteins. (**C**) PPI maps of the 25 core targets. (**D**) Results of GO analysis. (**E**) Bubble plots of KEGG analysis results.

A total of 243 targets of ginsenoside Rc were obtained from the PharmMapper and Super-PRED databases, and a total of 2243 targets of “chemotherapeutic intestinal mucositis” were obtained from the OMI and GeneCards databases. The Venn diagram (Figure 2A) represents the common targets of ginsenoside Rc and chemotherapeutic intestinal mucositis (CIM). The 95 targets displayed in the Venn diagram are considered potential targets for Rc in the treatment of CIM.

### 2.3. Construction of the PPI Network

The 95 potential targets were imported into the STRING database to obtain the PPI network. The PPI network was then visualized using Cytoscape 3.9.1, resulting in 95 nodes and 794 edges. As shown in Figure 2B, the larger the node, the higher its degree value. Then, a total of 25 core targets were screened using values of Betweenness ≥29.86304677, Closeness ≥ 0.005747, and Degree ≥ 19 (Figure 2C).

### 2.4. GO Enrichment and KEGG Pathway Analyses

GO and KEGG analyses were performed based on the Metascape database. As shown in Figure 2D, the results of GO analysis included 363 biological processes (BPs), 49 cellular components (CCs), and 94 molecular functions (MFs). The top 10 BPs, CCs, and MFs in the enrichment results were visualized; BPs included negative regulation of apoptotic process, phosphorylation, and peptidyl-tyrosine phosphorylation, etc.; CCs included receptor complex, extrinsic component of cytoplasmic side of plasma membrane, cytoplasm, etc.; and MFs includes RNA polymerase II transcription factor activity, ligand-activated sequence-specific DNA binding, protein tyrosine kinase activity, trans-membrane receptor protein tyrosine kinase activity, and so on. KEGG pathway enrichment results are shown in the bubble graph in Figure 2E. The results suggest that Rc may exert a therapeutic effect on chemotherapeutic intestinal mucositis through Hepatitis B, the PI3K-Akt signaling pathway, the chemokine signaling pathway, and the NF-kappa B signaling pathway.

It is well known that activation of PI3K/Akt signaling is essential for limiting inflammatory responses. NF-κB serves as a downstream signal of the PI3K/Akt signaling pathway, activation of which can inhibit the signaling of NF-κB, which can collectively exert an inhibitory effect on inflammation [13]. PI3K-AKT and NF-κB are also closely related to apoptosis.

### 2.5. Molecular Docking Validation Results

**Figure 3:**
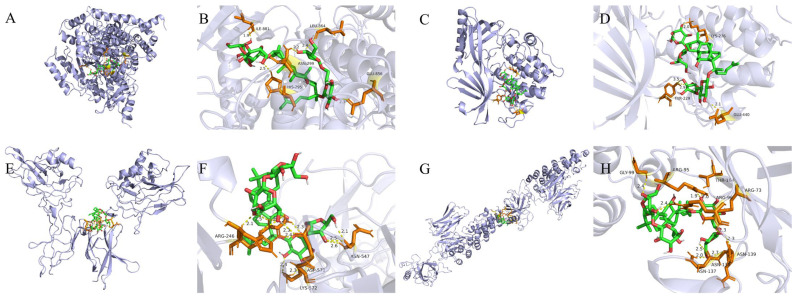
Molecular docking results. (**A**,**B**) Ginsenoside Rc and PI3K; (**C**,**D**) ginsenoside Rc and AKT; (**E**,**F**) Ginsenoside Rc and NF-κB p65; (**G**,**H**) Ginsenoside Rc and IκBα.

Key targets in the PI3 K-AKT and NF-κB signaling pathways were selected for molecular docking validation. The molecular docking results are shown in Figure 3.

It is well known that the lower the binding energy, the higher the affinity between receptor and ligand and the more stable the conformation. It is generally accepted that a binding energy of less than −5 kcal/mol indicates good binding activity between ligand and receptor. As shown in Table 1, the molecular docking results indicate that the binding energies of all these compounds were less than −5 kcal/mol, suggesting that these compounds have a high affinity for proteins.

### 2.6. Effect of Ginsenoside Rc on Proliferation of IEC-6 Treated by 5-Fu

**Figure 4:**
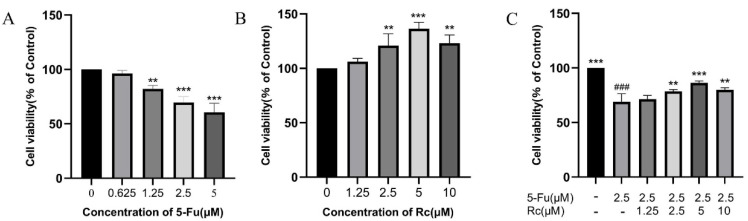
Effect of ginsenoside Rc on proliferation of IEC-6. (**A**) Effects of different concentrations of 5-Fu on IEC-6 cells. (**B**) Effects of different concentrations of Rc on IEC-6 cells. (**C**) Effects of different concentrations of Rc on IEC-6 cells after 5-Fu treatment. ** *p* < 0.01, *** *p* < 0.001 vs. model, ### *p* < 0.001 vs. control.

The effects of treatment with various concentrations of 5-Fu and Rc on IEC-6 cells were evaluated using CCK8. The results show that the viability of IEC-6 cells was all limited in the presence of 5-Fu, and the cell viability decreased to about 70% at 2.5 μM 5-Fu-treated cells (*p* < 0.001, Figure 4A). Therefore, 5-Fu at a concentration of 2.5 μM was chosen for subsequent experiments. We added 1.25, 2.5, 5, and 10 μM Rc to IEC-6 cells. Rc at all these concentrations increased IEC-6 cell survival, and the promotion of cell viability was most significant at 5 μM (*p* < 0.001, Figure 4B). Finally, IEC-6 cells with reduced cell viability induced by 2.5 μM 5-Fu were treated with different concentrations of Rc. The results show that 5 μM Rc significantly reversed the 5-Fu-induced reduction in IEC-6 cell viability (*p* < 0.001, Figure 4C).

### 2.7. Effect of Ginsenoside Rc on Permeability of IEC-6 Cells Treated by 5-Fu

**Figure 5:**
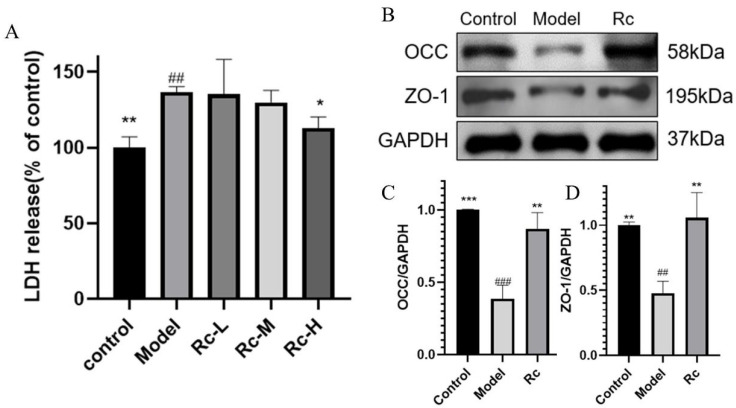
Effect of Rc on permeability of IEC-6 cells treated by 5-Fu. Among them, the model group indicates the treatment of IEC-6 cells with 2.5 μM 5-Fu. (**A**) LDH assay results. Rc-L, Rc-M, and Rc-H groups indicate the treatment of IEC-6 cells with 1.25 μM, 2.5 μM, 5 μM Rc, and 2.5 μM 5-Fu, respectively. (**B**) Bands of TJ proteins OCC and ZO-1. The original strips are shown in Figures S1 and S2 of the Supplementary file. The Rc group indicates the treatment of IEC-6 cells with 5 μM Rc and 2.5 μM 5-Fu. (**C**) Histogram comparing OCC protein expression. The Rc group indicates the treatment of IEC-6 cells with 5 μM Rc and 2.5 μM 5-Fu. (**D**) Comparison of histograms of ZO-1 protein expression. The Rc group indicates the treatment of IEC-6 cells with 5 μM Rc and 2.5 μM 5-Fu. * *p* < 0.05, ** *p* < 0.01, *** *p* < 0.001 vs. model, ## *p* < 0.01, ### *p* < 0.001 vs. control.

LDH, a marker of cell membrane permeability, flows out of the cell when the integrity of the cell membrane is disrupted, and permeability is increased, indicating that the intestinal epithelial barrier is damaged. As shown in Figure 5A, the LDH assay results indicated that the LDH release in the model group was significantly higher than that in the control group (*p* < 0.01, Figure 5A), but this trend was significantly improved by the addition of 5 μM Rc (*p* < 0.05, Figure 5A).

In addition, Western blotting results showed that the expression levels of two tight junction proteins (TJ proteins), OCC (*p* < 0.001, Figure 5C) and ZO-1 (*p* < 0.01, Figure 5D), were significantly downregulated after 5-Fu stimulation, and that the presence of 5 μM Rc resulted in their upregulation (*p* < 0.01, Figure 5C,D). All of these results suggested that Rc significantly ameliorated the 5-Fu-induced increase in IEC-6 cell permeability, thereby protecting the integrity of the intestinal epithelial cell barrier.

### 2.8. Ameliorative Effect of Ginsenoside Rc on 5-Fu-Induced Inflammation

**Figure 6:**
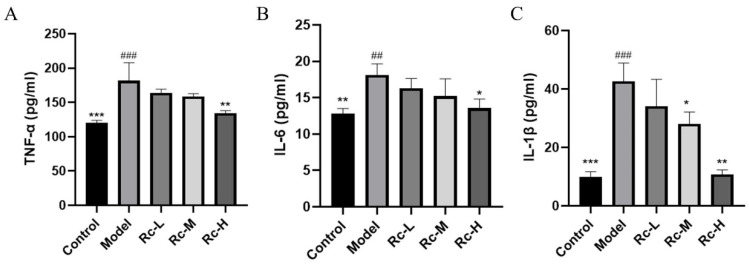
Ameliorative effect of ginsenoside Rc on 5-Fu-induced inflammation. Among them, the model group indicates the treatment of IEC-6 cells with 2.5 μM 5-Fu. Rc-L, Rc-M, and Rc-H groups indicate the treatment of IEC-6 cells with 1.25 μM, 2.5 μM, 5 μM Rc, and 2.5 μM 5-Fu, respectively. (**A**) Inflammatory factor kit results, including TNF-α. (**B**) Inflammatory factor kit results, including IL-6. (**C**) Inflammatory factor kit results, including IL-1β. * *p* < 0.05, ** *p* < 0.01, *** *p* < 0.001 vs. model, ## *p* < 0.01, ### *p* < 0.001 vs. control.

The results of the inflammatory factor kit experiment showed that 5-Fu significantly increased the levels of inflammatory cytokines, including TNF-α, IL-6, and IL-1β (*p* < 0.01, Figure 6A–C). Compared with the model group, the Rc-H group significantly downregulated the levels of the above inflammatory factors (*p* < 0.05, Figure 6A–C). This suggested that Rc significantly ameliorated 5-Fu-induced inflammation.

### 2.9. Effect of Ginsenoside Rc on IEC-6 Apoptosis

**Figure 7:**
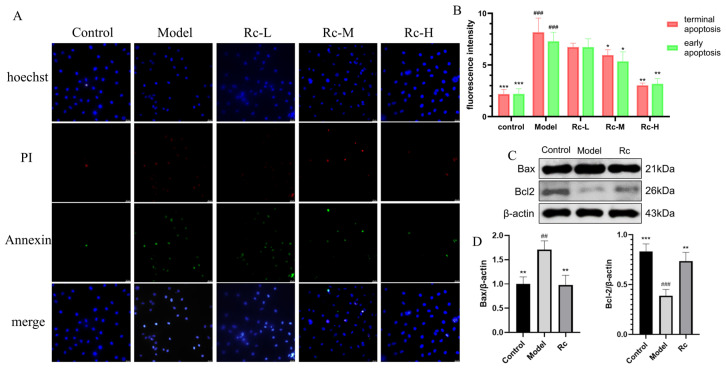
Effect of Rc on apoptosis after 5-Fu treatment. (**A**) Annexin V-FITC/PI Apoptosis Kit results, Rc-L, Rc-M, and Rc-H groups indicate the treatment of IEC-6 cells with 1.25 μM, 2.5 μM, 5 μM Rc, and 2.5 μM 5-Fu, respectively. (**B**) Histograms of early and late apoptosis in different groups. (**C**) Bands of apoptosis-related proteins Bax and Bcl-2. The original strips are shown in Figures S3 and S4 of the Supplementary File. The control group indicates cells without any reagent treatment; the Model group indicates cells treated with 2.5 μM 5-Fu only; and the Rc group indicates cells treated with 5 μM Rc and 2.5 μM 5-Fu. (**D**) Histograms comparing the expression of apoptosis-related proteins Bax and Bcl-2. * *p* < 0.05, ** *p* < 0.01, *** *p* < 0.001 vs. Model, ## *p* < 0.01, ### *p* < 0.001 vs. control.

Analysis with the Annexin V-FITC/PI Apoptosis Kit showed that the apoptosis rate significantly increased in the model group compared with the control group (*p* < 0.001, Figure 7B) for both early apoptosis (indicated by green fluorescence) and late apoptosis (indicated by red fluorescence). In contrast, treatment of 5-Fu-induced apoptotic cells with 5 μM Rc resulted in a significant reduction in apoptosis compared with the model group (*p* < 0.01, Figure 7B). Our findings suggested that Rc could attenuate 5-Fu-induced apoptosis.

As was observed from Figure 7C,D, the expression of pro-apoptotic protein Bax was significantly increased in the model group (*p* < 0.01, Figure 7D), and the expression of anti-apoptotic protein Bcl-2 was significantly decreased in the model group compared with the control group (*p* < 0.001, Figure 7D), which was consistent with the results of the Annexin V-FITC/PI Apoptosis Kit. The intervention of Rc significantly reversed this trend, suggesting that Rc significantly ameliorated 5-Fu-induced apoptosis in IEC-6 cells.

### 2.10. Effect of Ginsenoside Rc on Protein Expressions of PI3K/AKT and NF-κB Pathway

**Figure 8:**
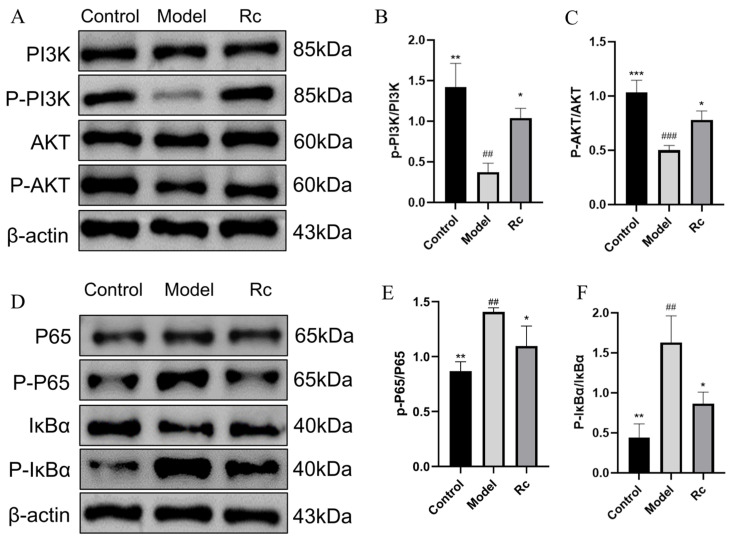
Effect of Rc on protein expressions in PI3K/AKT and NF-κB pathways. Among them, the model group indicates the treatment of IEC-6 cells with 2.5 μM 5-Fu. The Rc group indicates the treatment of IEC-6 cells with 5 μM Rc and 2.5 μM 5-Fu. (**A**) Bands of proteins in the PI3K/AKT signaling pathway. The original strips are shown in Figures S5–S8 of the Supplementary file. (**B**) Histogram comparing p-PI3K/PI3K protein expression in each group. (**C**) Comparison of histograms of p-AKT/AKT protein expression. (**D**) Bands of proteins in the NF-κB signaling pathway. The original strips are shown in Figures S9–S12 of the Supplementary file. (**E**) Histograms comparing p-P65/P65 protein expression in each group. (**F**) Histograms comparing p-IκBα/IκBα protein expression. * *p* < 0.05, ** *p* < 0.01, *** *p* < 0.001 vs. model, ## *p* < 0.01, ### *p* < 0.001 vs. control.

As shown in Figure 8, p-PI3K/PI3K and p-AKT/AKT levels were significantly increased in the Rc group compared with the Model group (*p* < 0.05, Figure 8B,C). As shown in Figure 8D–F, the levels of P65 and p-IκB/IκB were significantly lower than those in the model group (*p* < 0.05 Figure 8E,F). This suggests that Rc activates the PI3K/AKT signaling pathway and inhibits the NF-κB signaling pathway.

### 2.11. Effects of Ginsenoside Rc on mRNA Expressions of PI3K-AKT/NF-κB Pathway

**Figure 9:**
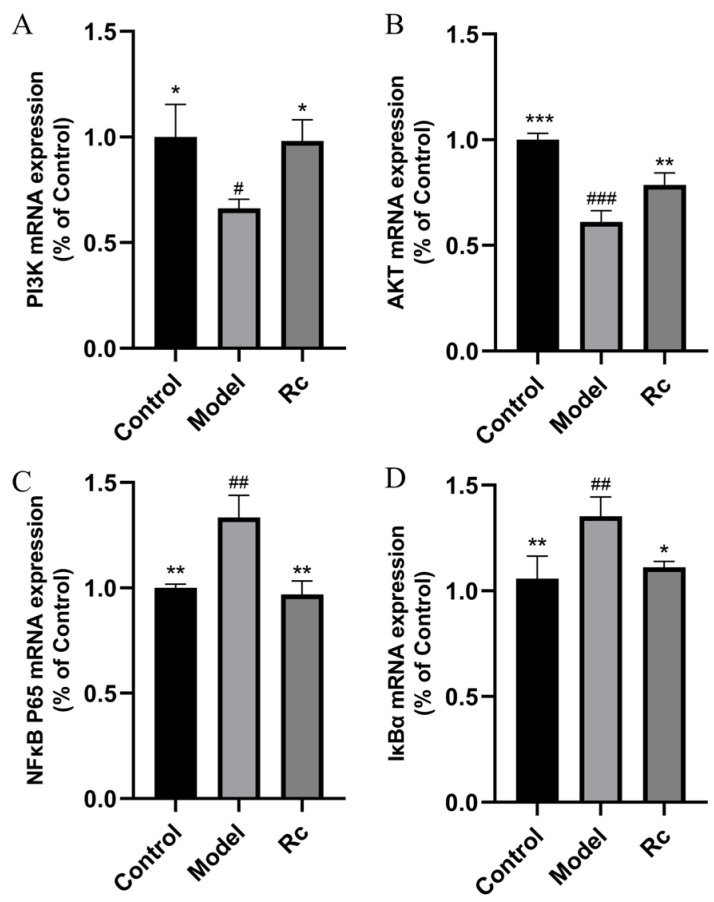
Effects of ginsenoside Rc mRNA expressions of PI3K-AKT/NF-κB pathway. Among them, the model group indicates the treatment of IEC-6 cells with 2.5 μM 5-Fu. The Rc group indicates the treatment of IEC-6 cells with 5 μM Rc and 2.5 μM 5-Fu. (**A**) mRNA expressions of *PI3K*. (**B**) mRNA expressions of *AKT*. (**C**) mRNA expressions of *NF-κB P65*. (**D**) mRNA expressions of *IκBα*. * *p* < 0.05, ** *p* < 0.01, *** *p* < 0.001, # *p* < 0.05 vs. model, ## *p* < 0.01, ### *p* < 0.001 vs. control.

The mRNA expression related to the PI3K-AKT signaling pathway was significantly reduced in the model group compared to the control group (*p* < 0.05, Figure 9) and was significantly increased by the addition of Rc (*p* < 0.05, Figure 9). These results further confirmed that Rc could inhibit the PI3K-AKT signaling pathway at the gene level.

mRNA expressions of *NF-κB P65* and *IκBα* was significantly increased in the model group compared with the control group (*p* < 0.05, Figure 9). The expression of these mRNAs associated with the NF-κB signaling pathway was significantly reduced after Rc intervention (*p* < 0.05, Figure 9).

## 3. Discussion

5-Fu is a popular chemotherapeutic drug, but it can cause damage to the gastrointestinal tract, leading to CIM [14]. Rc has been reported to possess antioxidant and anti-inflammatory activities [15]. It has also been found to attenuate DSS-induced ulcerative colitis, intestinal inflammation, and barrier function [12]. IEC-6 cells have been widely used in the study of intestinal epithelial barrier damage and intestinal inflammation [16,17,18]. Therefore, we chose 5-Fu as the model drug and selected IEC-6 cells for subsequent experiments to investigate the therapeutic effect of Rc against 5-Fu-induced chemotherapeutic intestinal mucositis.

Chemotherapeutic drugs can disrupt the intestinal epithelial barrier and permeability [19]. Disruption of the intestinal epithelial barrier is one of the characteristics of chemotherapeutic intestinal mucositis. In our in vivo study, 20 mg·kg^−1^ Rc significantly ameliorated 5-Fu-induced body weight loss and diarrhea scores in ICR mice. Ginsenoside Rc also significantly ameliorated morphological injury, including epithelial vacuolization, inflammatory cell infiltration, mucosal defects, and crypt damage, as well as reduced intestinal permeability in mice. In our in vitro experiments, Rc significantly reduced the release of LDH in IEC-6 cells. Western blotting results also showed that the presence of Rc significantly increased the levels of OCC and ZO-1. LDH can be used to assess cell membrane permeability and integrity because it does not leak out when the cell membrane is intact [20]. ZO-1 and OCC are TJ proteins, which are apical components of the intestinal epithelial barrier that maintain barrier integrity [21]. The above results indicate that Rc had a therapeutic effect on 5-Fu-induced CIM mice and also reduced cell membrane permeability and exerted a protective effect on intestinal epithelial cells. However, the underlying mechanism of Rc remains unclear.

Network pharmacology was applied to search for possible pathways by which Rc could improve CIM in mice. The results suggest that the protective effect of Rc on CIM might be related to the PI3K-AKT and NF-κB signaling pathways. In vitro cellular experiments and molecular biological methods were subsequently performed to validate the signaling pathways.

Apoptosis and inflammatory response are recognized as key events in the pathogenesis of chemotherapeutic intestinal mucositis [22,23,24]. Activation of PI3K-Akt signaling is critical for the inhibition of apoptosis and inflammatory responses and has long been recognized as a negative regulator of NF-κB signaling as a major downstream effector of PI3K [25,26,27]. We used the Annexin V-FITC/PI Apoptosis Kit and a Western blotting assay to explore the effect of Rc on apoptosis and an ELISA kit to detect the effect of Rc on inflammatory response. AKT activation can inhibit the NF-κB pathway, which reduces the expression level of Bax/Bcl-2 and ultimately leads to the inhibition of apoptosis [28,29], which is consistent with our experimental results. In NF-κB signaling, activation of IKK further phosphorylates the IκB protein and releases the NF-κB complex p50-p65 [13]. NF-κB then translocates to the nucleus, where it regulates the expression of pro-inflammatory factors such as TNF-α, IL-6, and IL-1β [30,31,32]. The ELISA kit results showed that the overgeneration of TNF-α, IL-6, and IL-1β induced by 5-Fu was significantly restored to near-normal levels by Rc. We suggest that the action of Rc may be selective, inhibiting the expression of inflammatory factors by activating the anti-inflammatory branch of the PI3K/Akt pathway, inhibiting the activation of IKK/NF-κB or enhancing the stabilization of IκBα, and at the same time decreasing the transcriptional activity of NF-κB [13]. This result reflects the complex mechanism of Rc in the regulation of inflammation, although further studies are needed to elucidate its specific mode of action.

In addition, the Western blot results show that the total protein expression levels of PI3K, Akt, NF-kB (p65), and IκBα were not significantly affected by 5-FU or Rc treatment. However, qPCR results (Figure 9) revealed that their mRNA levels were modulated. This discrepancy suggests that the PI3K/Akt and NF-kB signaling pathways are predominantly regulated at the post-translational level. Mechanisms such as kinase-mediated activation of PI3K/Akt, ubiquitin–proteasome system (UPS)-mediated degradation of IκBα, release of p65 from IκBα, and nuclear translocation of free p65 may play a crucial role. It is well-established that the PI3K/Akt and NF-kB pathways are tightly regulated through post-translational mechanisms, including phosphorylation, ubiquitination, and proteasomal degradation. These processes allow rapid and dynamic modulation of signaling in response to cellular stimuli, which may explain the observed differences between protein and mRNA expression levels in our study [33,34,35].

Our study suggests that Rc may bind to PI3K and activate the PI3K/Akt pathway. However, Rc treatment significantly reduced LDH release (Figure 5), which appears to be inconsistent with the well-established mechanism by which PI3K/Akt activation promotes HIF-1α expression and, subsequently, LDH production. This apparent discrepancy can be explained by considering the broader regulatory effects of Rc on multiple pathways. The modulation of the PI3K/Akt signaling pathway by Rc may be selective, i.e., Rc activates branching pathways associated with cell survival and anti-inflammation while inhibiting HIF-1α-related metabolic pathways [34]. It is also possible that the antioxidant and membrane-protective properties of Rc attenuated cellular damage, thereby reducing LDH release [36].

Although the results of this study demonstrate that ginsenoside Rc provides significant protective effects against 5-Fu-induced CIM, there are several limitations to be considered. First, the study was primarily based on animal models and in vitro experiments, and its clinical efficacy remains to be further validated. Additionally, a clearer understanding of the specific molecular targets and interactions of Rc with the signaling pathways requires further in-depth mechanistic studies.

**Figure 10:**
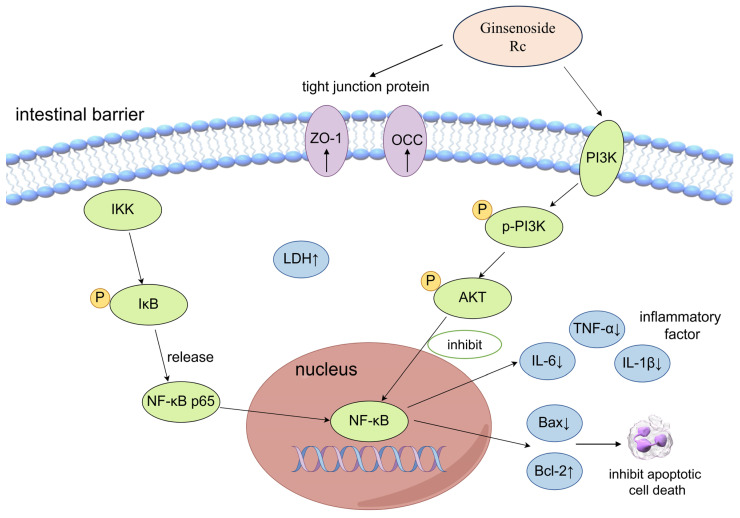
Potential mechanism of action of Rc on 5-Fu-induced CIM.

In conclusion, this study suggests that ginsenoside Rc significantly alleviated 5-Fu-induced CIM through anti-inflammatory and anti-apoptotic pathways, potentially mediated by the regulation of the PI3K-AKT and NF-κB signaling pathways. The potential mechanism of action of Rc on 5-Fu-induced CIM is shown in Figure 10. These findings provide experimental evidence for Rc as a potential therapeutic agent for CIM and offer new insights into its applications in anti-inflammatory and anticancer therapies.

## 4. Materials and Methods

### 4.1. Chemicals and Reagents

Ginsenoside Rc was purchased with a purity higher than 98% from Chengdu Pufide Controls Technology Co. (Chengdu, China). The 5-fluorouracil injection was purchased from Tianjin Jin Yao Pharmaceutical Co. (Tianjin, China). Loperamide hydrochloride capsules (LOP) were purchased from Xi’an Janssen Pharmaceutical Co. (Batch No. NAJ6932) (Xi’an, China). Small intestine crypt epithelial cell line IEC-6 was purchased from Shanghai Junmai Biotechnology Co. (Shanghai, China).

### 4.2. In Vivo Experiments on Animals

#### 4.2.1. Animals and Experimental Design

Specific pathogen-free (SPF) grade male ICR mice (18–20 g) were obtained from Chengdu Dashi Experimental Animal Co., Ltd. (Chengdu, China). The Animal Ethics Committee of Chengdu University of Traditional Chinese Medicine (No. 2024034) approved all protocols. The mice were housed under standard environmental conditions, with a temperature of 23 ± 2 °C, humidity of 50% ± 5%, and a 12 h light/dark cycle. They were provided with a standard diet and water for 3 days to acclimate to the new conditions prior to the experiments.

**Figure 11:**
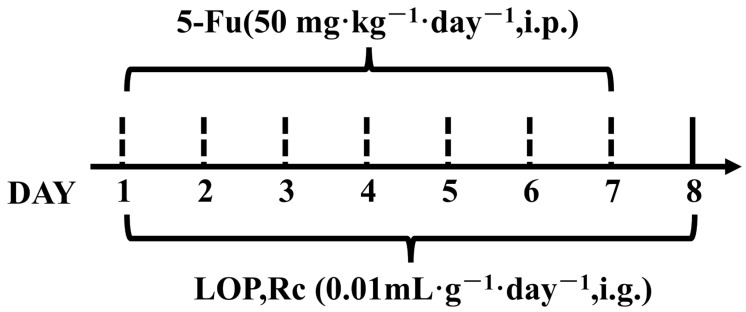
Experimental cycle of CIM due to Rc treatment with 5-Fu (*n* = 6).

A total of 30 mice were randomly divided into five groups: control, model, LOP (positive group) (3 mg·kg^−1^), Rc-L (10 mg·kg^−1^), and Rc-H (20 mg·kg^−1^), with six mice in each group. LOP and Rc were dissolved in a 0.3% CMC-Na solution and stored at 4 °C. The animal experiment protocol is shown in Figure 11. Mice in the LOP and treatment groups (Rc-L and Rc-H) received LOP and varying concentrations of Rc at a dose of 0.01 mL·g^−1^·day^−1^ for 8 days. Meanwhile, an equivalent volume of CMC-Na solution was administered to the control and model groups. The LOP and dosing groups were modeled by administering 50 mg·kg^−1^·day^−1^ of 5-Fu from day 1 to day 7.

#### 4.2.2. Recording Weight and Diarrhea Extent

During the course of the experiment, the body weight and diarrhea of the experimental mice were recorded daily at a fixed time and place. Diarrhea was evaluated based on Akinobu Kurita’s method [37]: 0, no diarrhea or normal stools; 1, mild diarrhea with wet, soft stools; 2, moderate diarrhea with loose stools and mild perianal stains; 3, severe diarrhea with watery stools and severe perianal stains.

#### 4.2.3. Histopathologic Evaluation

Approximately 2 cm of distal ileum was taken from mice, fixed in 4% paraformaldehyde, and then embedded in paraffin, and the tissues were cut into 4 μm sections, which were then stained with hematoxylin–eosin (HE) for histopathological evaluation. Images of the stained sections were captured by TEM (JEM-1400-FLASH).

#### 4.2.4. Detection of Intestinal Permeability in Mice

Serum levels of diamine oxidase (DAO) and D-(-)-lactic acid (D-LA) were assayed in mice in each group according to the instructions of the reagent vendors.

### 4.3. Network Pharmacology and Molecular Docking

#### 4.3.1. Ginsenoside Rc Target Prediction

The potential targets of ginsenoside Rc (CAS No. 11021-14-0) were predicted using two target identification tools: PharmMapper (http://lilab-ecust.cn/pharmmapper/, accessed on 20 May 2024) and Super-PRED (https://prediction.charite.de/, accessed on 20 May 2024). The outputs from both tools were collected, and target protein names were converted to gene symbols using the UniProt database (https://www.uniprot.org/, accessed on 20 May 2024). The resulting gene lists from the two tools were merged, and duplicate targets were removed. This process generated a final list of predicted targets for ginsenoside Rc.

#### 4.3.2. Screening of CIM-Related Targets

To identify the target genes related to chemotherapeutic intestinal mucositis (CIM), two publicly available databases were used: OMIM (Online Mendelian Inheritance in Man) (https://www.omim.org/, accessed on 20 May 2024) and GeneCards (https://www.genecards.org/, accessed on 20 May 2024). The lists of genes obtained from OMIM and GeneCards were combined, and duplicate genes were removed. The resulting gene set represented the pool of CIM-related targets.

#### 4.3.3. Construction of a Protein–Protein Interaction (PPI) Network

Cross-targets of CIM-related genes with predicted Rc targets were obtained and visualized by constructing Venn diagrams using Venny 2.1.0 (https://bioinfogp.cnb.csic.es/tools/venny/index.html, accessed on 21 May 2024). The intersecting genes represented the potential targets of Rc for CIM treatment. The cross-targets were extracted into the STRING database (https://string-db.org, accessed on 21 May 2024) to construct a PPI network. The species was selected as “Homo sapiens”. Finally, the PPI results were imported into Cytoscape 3.8.0 software to construct the PPI network.

#### 4.3.4. GO Enrichment and KEGG Pathway Analysis

The core targets of ginsenoside Rc for the treatment of CIM were imported into the Metascape (http://metascape.org/, accessed on 27 May 2024) database, and the species was selected as “Homo sapiens”. Gene Ontology (GO) and Kyoto Encyclopedia of Genes and Genomes (KEGG) enrichment analyses were selected to evaluate the crossover genes, with the FDR < 0.05 and *p* < 0.05.

#### 4.3.5. Molecular Docking Validation

Ten potential target proteins and Rc were molecularly docked. The 3D structures of the proteins were retrieved from Protein Data Bank (PDB) (http://www.rcsb.org/, accessed on 30 May 2024) and then imported into the software Pymol 1.5.7 by removing water molecules and primitive ligands, and then the target proteins were imported into AutoDock Tools 1.5.7 for hydrogenation, charge calculation, and nonpolar hydrogen combination. The results were then stored in PDBQT format. The 2D structures of Rc small molecules were obtained via PubChem (https://pubchem.ncbi.nlm.nih.gov/, accessed on 30 May 2024). Finally, molecular docking was performed using AutoDock Vina 1.5.7, and the results were visualized using PyMOL 1.5.7.

### 4.4. In Vitro Experimental Validation

#### 4.4.1. Cell Culture

IEC-6 cells were incubated using a DMEM medium containing 10% FBS, 100 U/mL penicillin, and 0.1 mg/mL streptomycin. IEC-6 cells were incubated at 37 °C in a humidified 5% CO_2_ incubator. The medium was changed every 2–3 days during cell growth.

#### 4.4.2. Cell Viability Assay

IEC-6 cells were homogeneously inoculated into 96-well plates at a density of 5×10^3^ cells/well, and the cells were incubated using complete medium for 24 h. The medium was changed after 24 h, and the incubation was continued for 24 h using medium containing the drug. In the blank group (no cells), only an equal amount of medium was added; in the control group (with cells), an equal amount of drug carrier solvent was added (dimethylsulfoxide content <0.1%); model group: 5-Fu (0.625, 1.25, 2.5, 5 μM); administration group: Rc (1.25, 2.5, 5, 10 μM). A total of 10 μL of the drug was added to each well after 24 h. Cell Counting Kit-8 (CCK-8) reagent was added for 1 h, and then absorbance was read at 450 nm using a spectrophotometer.

#### 4.4.3. Measurement of LDH Release

IEC-6 cells (5000 cells/well) were inoculated into 96-well plates and incubated under a cell culture incubator containing 5% CO_2_ at 37 °C for 24 h. Cells were then treated with different concentrations of ginsenoside Rc (1.25, 2.5, and 5 μM) and 2.5 μM 5-Fu for 24 h. Finally, LDH release was performed using an LDH detection kit according to the manufacturer’s instructions.

#### 4.4.4. Enzyme-Linked Immunosorbent Assay (ELISA)

IEC-6 cells were inoculated into 6-well plates and administered according to Section 4.4.2 after 24 h. After another 24 h, the cell supernatant was collected, and TNF-α, IL-6, and IL-1β were detected according to the ELISA kit.

#### 4.4.5. Apoptosis Assay

IEC-6 cells were inoculated into 6-well plates and incubated for 24 h at 37 °C under a cell culture incubator containing 5% CO_2_. The cells were then treated with different concentrations of ginsenoside Rc (1.25, 2.5, and 5 μM) and 2.5 μM of 5-Fu for 24 h. Finally, apoptosis staining was performed according to the instructions of the Annexin V-FITC/PI Apoptosis Kit (Elabscience, Chengdu, China), followed by the use of Image Xpress Micro Confocal (Molecular Devices, Shanghai, China) to obtain fluorescence images. Early apoptotic cells showed green fluorescence and late apoptotic cells showed red fluorescence.

#### 4.4.6. Western Blotting (WB)

IEC-6 cells were inoculated in 6-well plates, and cells were treated according to Section 4.4.2 after 24 h. After washing the IEC-6 cells three times using cold PBS, the cells were lysed on ice with Ripa Lysis Buffer. The Bicinchoninic Acid Assay (BCA) protein assay kit was used to quantify the protein concentration of each sample according to the manufacturer’s instructions. Based on the BCA quantification results, Sodium Dodecyl Sulfate-Polyacrylamide Gel Electrophoresis (SDS-PAGE) gels were prepared, and then the proteins were transferred to PVDF membranes. For occlusion, 5% BSA was used for two hours, followed by the use of a cell membrane containing Occudin (OCC), Zonula Occludens-1 (ZO-1), B-cell lymphoma 2 (Bcl-2), Bcl-2-associated X protein (Bax), Phosphoinositide 3- kinase (PI3K), Protein Kinase B (AKT), Phosphorylated Phosphoinositide 3-kinase (p-PI3K), Phosphorylated Protein Kinase B (p-AKT), Inhibitor of Nuclear Factor kappa-Bα (IκBα), Phosphorylated Inhibitor of Nuclear Factor kappa-B (p-IκBα), Nuclear Factor kappa-B p65 Subunit (P65), and Primary Antibody of Phosphorylated Nuclear Factor kappa-B p65 Subunit (p-P65). The cells were then incubated overnight. The next day, after washing with Tris-buffered Saline with Tween 20 (TBST), the protein bands were incubated with a secondary antibody for 1.5 h. An Enhanced Chemiluminescence Kit was used to visualize the protein bands. Finally, the chemiluminescence results were analyzed using Image-J 1.54 software.

#### 4.4.7. Real-Time Quantitative PCR (RT-PCR)

RNA was reverse transcribed to cDNA using RT Easy^TM^II (with gDNase) (FOREGENE, Beijing, China) according to the reagent vendor’s instructions. RT-PCR was performed using RT-PCR Easy^TM^-SYBR Green I (FOREGENE, China) and Archimed X4 system (ROCGENE, Shanghai, China). RT-PCR reaction conditions were as follows: 95 °C for 30 s, 95 °C for 5 s, and 60 °C for 30 s for 40 cycles. The primer sequences used for the study are shown in Table 2. Finally, mRNA expression was determined using the 2^−ΔΔCt^ method.

### 4.5. Statistical Analysis

All experimental results were processed and analyzed using GraphPad Prism 9.5, and comparisons of multiple samples were made using one-way ANOVA followed by Dunnett’s test or Tukey’s test; *p*-value < 0.05 was considered statistically significant.

## 5. Conclusions

This study demonstrates that Rc exerts significant protective effects against 5-Fu-induced CIM in vivo and in vitro. In vivo experiments have shown that Rc could effectively alleviate the symptoms of CIM in mice, including increasing body weight, improving diarrhea, and protecting against intestinal damage. In addition, ginsenoside Rc significantly reduced cell death and apoptosis, protected the integrity of the intestinal barrier and inhibited the overproduction of pro-inflammatory cytokines through the PI3K-AKT/NF-κB pathway. In conclusion, Rc has shown therapeutic potential as an anti-CIM agent.

## Figures and Tables

**Table 1:** Docking results of ginsenoside Rc with key target molecules.

Target	PDB ID	Binding Energy (kcal/mol)	Center_x	Center_y	Center_z
PI3K	3APD	−9.7	32.458	3.119	25.71
AKT	6HHG	−7.8	14.305	−13.778	−14.633
NF-κB p65	1VKX	−7.1	1.153	40.241	56.792
IκBα	1NFI	−9.2	−5.372	62.732	45.296

**Table 2:** Primer sequences used in this experiment.

Gene	Forward	Reverse
*PI3K*	AACCGGGACAGCTAAGCAAC	TCCCGGCTTCATTCACCTCC ACTCGTTCATGGTCACACGG
*AKT*	GAGACGATGGACTTCCGGTC	
*NF-κB p65*	TTCAACATGGCAGACGACGA	AGGTATGGGCCATCTGTTGAC
*IκBα*	GAATCCTGACCTGGTCTCGC	CAGTCATCGTAGGGCAACTCA
*β-actin*	CCTTCCTGGGCATGGAGTC	TGATCTTCATTGTGCTGGGTG

## Data Availability

The original contributions presented in this study are included in the article/supplementary material. Further inquiries can be directed to the corresponding author.

## Supplementary Materials

The following supporting information can be downloaded at: https://www.mdpi.com/article/10.3390/ijms252313085/s1.

## Abbreviations

5-Fu, 5-Fluorouracil; Rc, ginsenoside Rc; IEC, Intestinal epithelial cells; CIM, chemotherapeutic intestinal mucositis; DSS, dextran sulfate sodium; LOP, Loperamide hydrochloride capsules; HE, hematoxylin–eosin; DAO, diamine oxidase; D-LA, D-(-)-lactic acid; PPI, protein–protein interaction; GO, Gene Ontology; KEGG, Kyoto Encyclopedia of Genes and Genomes; CCK-8, Cell Counting Kit-8; LDH, lactate dehydrogenase; ELISA, Enzyme-linked immunosorbent assay; WB, Western blotting; OCC, Occudin; ZO-1, Zonula Occludens-1; Bcl-2, B-cell lymphoma 2; Bax, Bcl-2-associated X protein; PI3K, Phosphoinositide 3- kinase; AKT, Protein Kinase B; p-PI3K, Phosphorylated Phosphoinositide 3-kinase; p-AKT, Phosphorylated Protein Kinase B; IκB, Inhibitor of Nuclear Factor kappa-B; p-IκB, Phosphorylated Inhibitor of Nuclear Factor kappa-B; P65, Nuclear Factor kappa-B p65; BP, Biological process; CC, Cellular component; MF, Molecular Function; TJ, tight junction.
