# Supplementary material for: Ameliorative Effect of Ginsenoside Rc on 5-Fluorouracil-Induced Chemotherapeutic Intestinal Mucositis via the PI3K-AKT/NF-κB Signaling Pathway: In Vivo and In Vitro Evaluations
Source: Int J Mol Sci. 2024 Dec 5;25(23):13085. doi: 10.3390/ijms252313085 (PMC11642520; doi:10.3390/ijms252313085)
Supplement: Supplementary file 1 [file ijms-25-13085-s001.zip › ijms-3326130-supplementary.pdf]

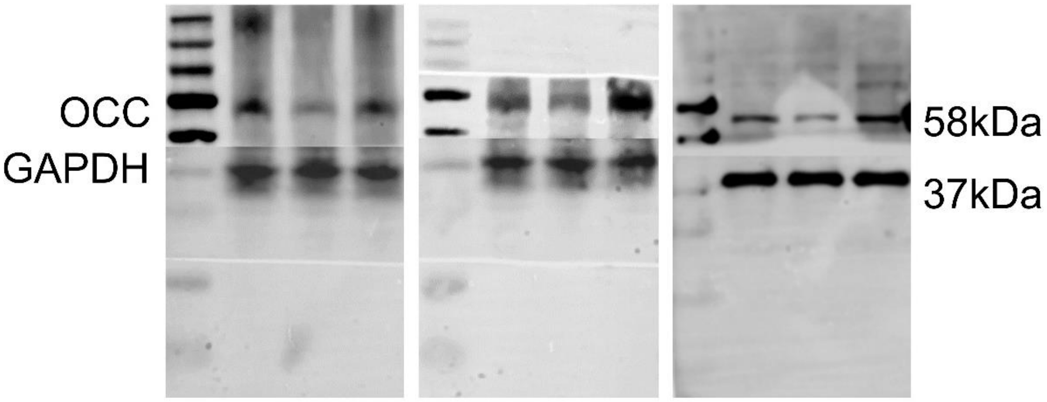

Figure S1 Western blot images for OCC. Bands from left to right are Control group, Model group and Rc group.

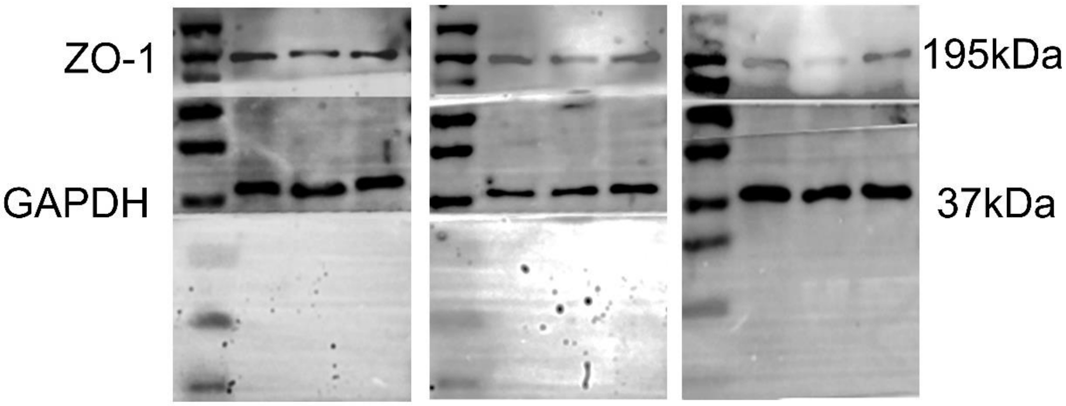

Figure S2 Western blot images for ZO-1. Bands from left to right are Control group, Model group and Rc group.

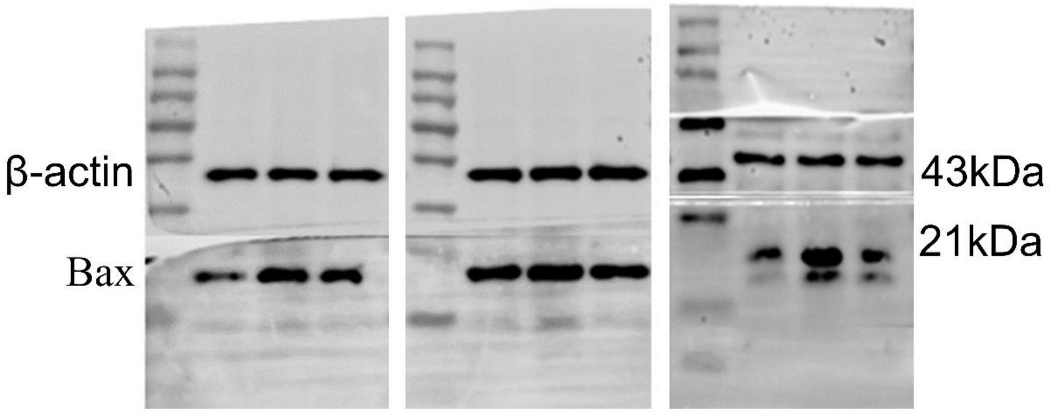

Figure S3 Western blot images for
Bax. Bands from left to right are Control group, Model group and Rc group.

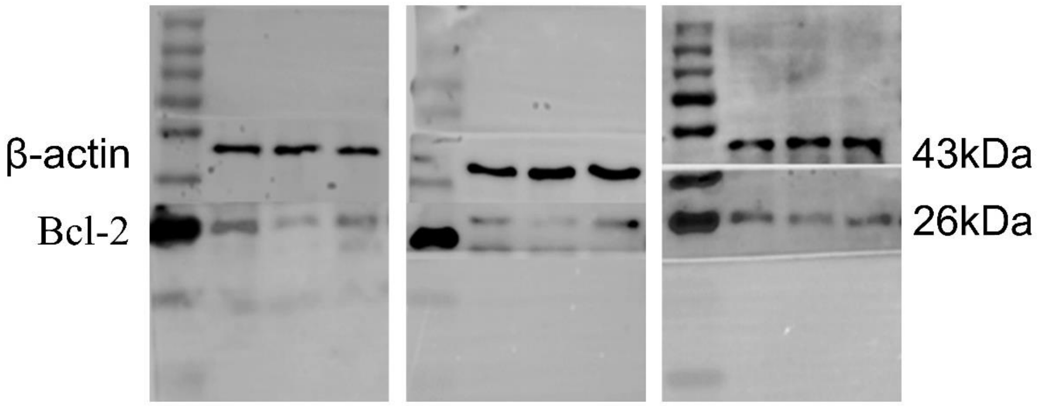

Figure S4 Western blot images for Bcl-2. Bands from left to right are Control group, Model group and Rc group.

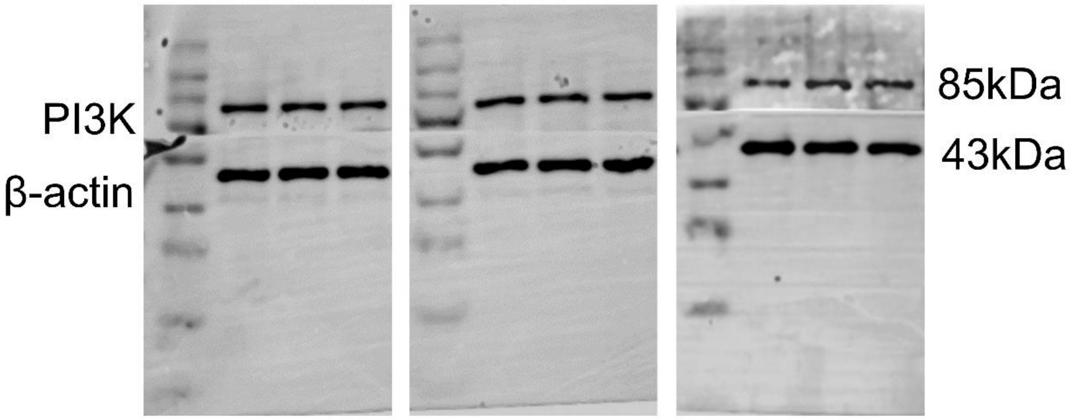

Figure S5 Western blot images for PI3K. Bands from left to right are Control group, Model group and Rc group.

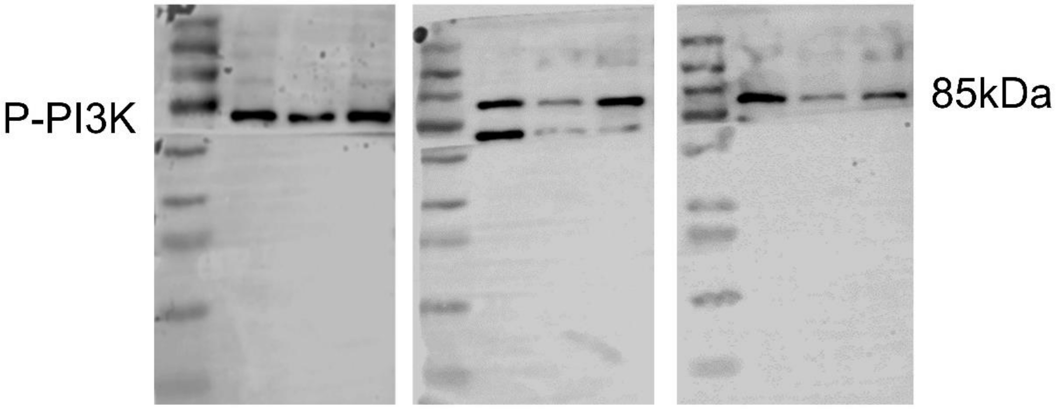

Figure S6 Western blot images for p-PI3K. Bands from left to right are Control group, Model group and Rc group.

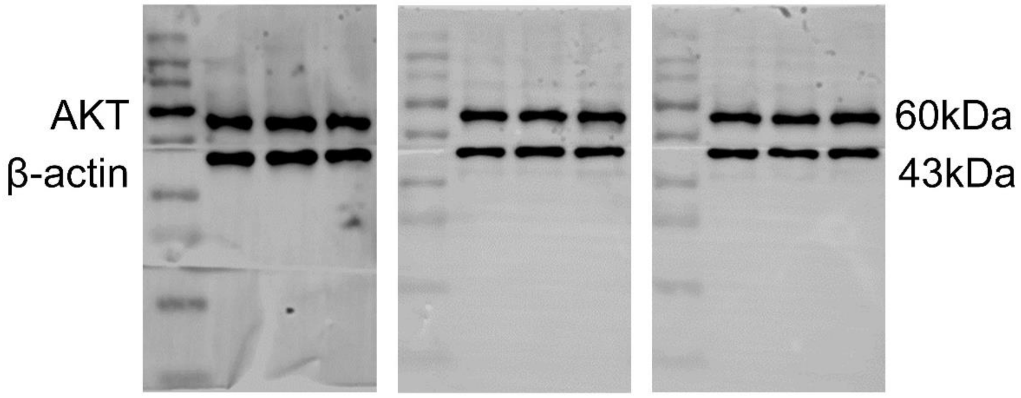

Figure S7 Western blot images for AKT. Bands from left to right are Control group, Model group and Rc group.

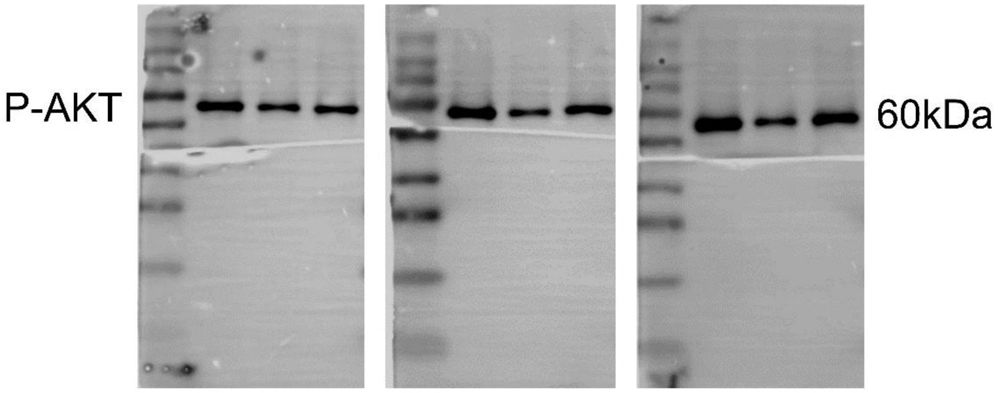

Figure S8 Western blot images for p-AKT. Bands from left to right are Control group, Model group and Rc group.

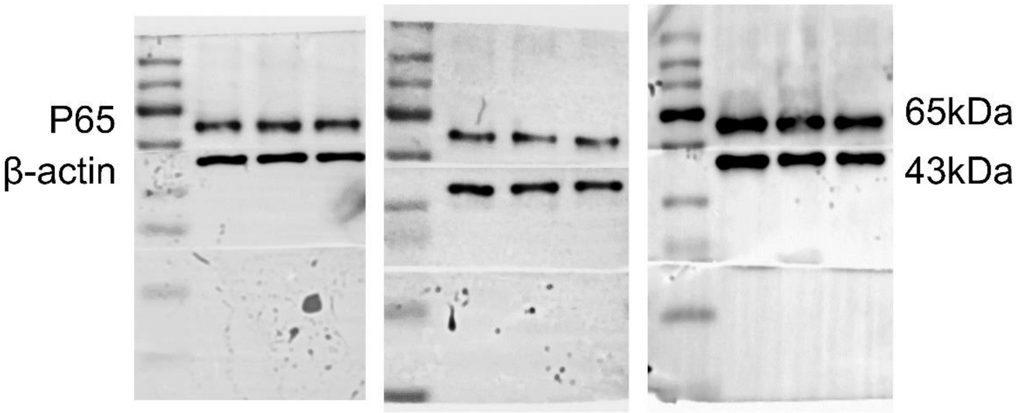

Figure S9 Western blot images for P65. Bands from left to right are Control group, Model group and Rc group.

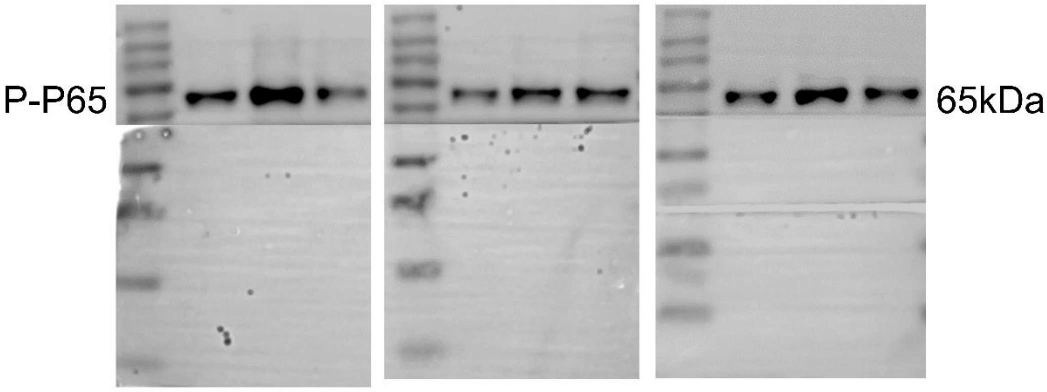

Figure S10 Western blot images for p-P65. Bands from left to right are Control group, Model group and Rc group.

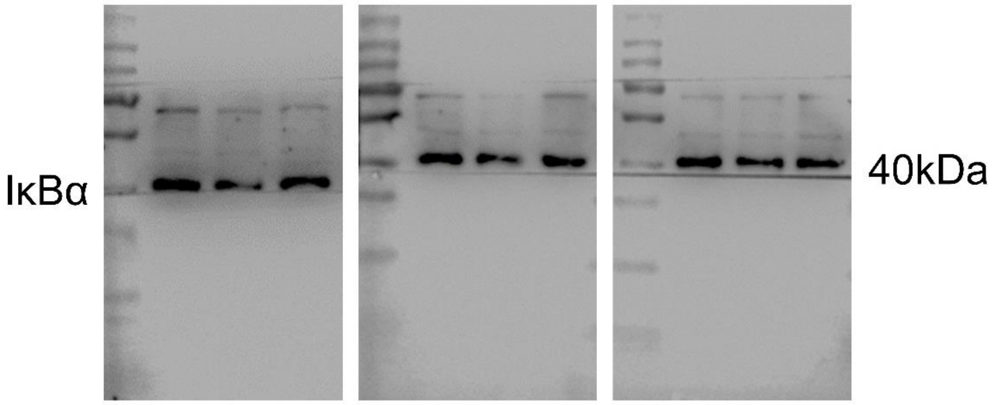

Figure S11 Western blot images for IκBα. Bands from left to right are Control group, Model group and Rc group.

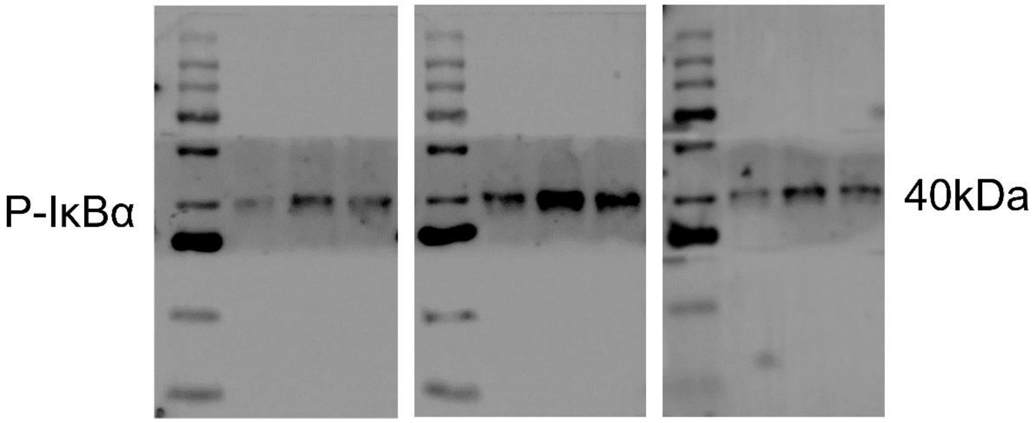

Figure S12 Western blot images for p-IκBα. Bands from left to right are Control group, Model group and Rc group.
